# Chemical Chaperone and Inhibitor Discovery: Potential Treatments for Protein Conformational Diseases

**DOI:** 10.4137/pmc.s212

**Published:** 2007-12-11

**Authors:** Jian-Hua Zhao, Hsuan-Liang Liu, Hsin-Yi Lin, Chih-Hung Huang, Hsu-Wei Fang, Shiao-Shing Chen, Yih Ho, Wei-Bor Tsai, Wen-Yih Chen

**Affiliations:** 1 Department of Chemical Engineering and Biotechnology, National Taipei University of Technology, 1 Sec. 3 ZhongXiao E. Rd., Taipei 10608; 2 Graduate Institute of Biotechnology, National Taipei University of Technology, 1 Sec. 3 ZhongXiao E. Rd., Taipei, Taiwan 10608; 3 Institute of Environmental Engineering and Management, National Taipei University of Technology, 1 Sec. 3 ZhongXiao E. Rd., Taipei, Taiwan 10608; 4 School of Pharmacy, Taipei Medical University, 250 Wu-Hsing St., Taipei, Taiwan 110; 5 Department of Chemical Engineering, National Taiwan University, 1 Sec. 4 Roosevelt Rd., Taipei, Taiwan 106; 6 Department of Chemical and Materials Engineering, National Central University, 300 Jhongda Rd., Jhongli City, Taoyuan County, Taiwan 32001

**Keywords:** misfolding, Alzheimer’s disease, Prion’s disease, Parkinson’s disease, Huntington’s disease, amyloid, chemical chaperone, molecular dynamics simulation, structure-based drug design, protein conformational disease

## Abstract

Protein misfolding and aggregation cause a large number of neurodegenerative diseases in humans due to (i) gain of function as observed in Alzheimer’s disease, Huntington’s disease, Parkinson’s disease, and Prion’s disease or (ii) loss of function as observed in cystic fibrosis and α1-antitrypsin deficiency. These misfolded proteins could either lead to the formation of harmful amyloids that become toxic for the cells or to be recognized and prematurely degraded by the protein quality control system. An increasing number of studies has indicated that some low-molecular-weight compounds named as chemical chaperones can reverse the mislocalization and/or aggregation of proteins associated with human conformational diseases. These small molecules are thought to non-selectively stabilize proteins and facilitate their folding. In this review, we summarize the probable mechanisms of protein conformational diseases in humans and the use of chemical chaperones and inhibitors as potential therapeutic agents against these diseases. Furthermore, recent advanced experimental and theoretical approaches underlying the detailed mechanisms of protein conformational changes and current structure-based drug designs towards protein conformational diseases are also discussed. It is believed that a better understanding of the mechanisms of conformational changes as well as the biological functions of these proteins will lead to the development and design of potential interfering compounds against amyloid formation associated with protein conformational diseases.

## Protein Conformational Diseases

Protein misfolding is believed to be the primary cause of several neurodegenerative diseases in humans, such as Alzheimer’s disease (AD), Creutzfeldt-Jakob disease, Gaucher’s disease, Huntington’s disease, Parkinson’s disease, Prion’s disease, cystic fibrosis (CF), α1-antitrypsin deficiency, and many others. In most of the cases, protein misfolding takes place due to an undesirable mutation in the polypeptide chain, an unfavorable physiological environment or, in a few cases, some less known reasons.

**Figure 1.**
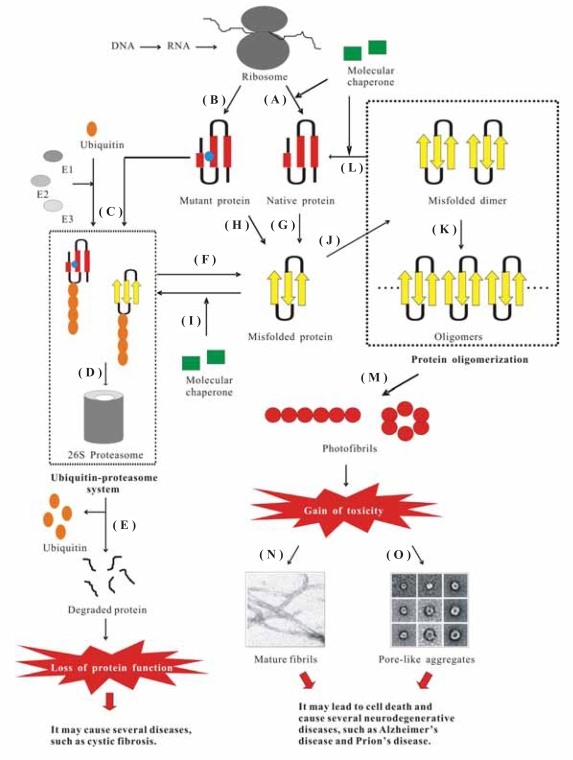
The pathway of protein synthesis and degradation in the cell. (**A**) Nascent polypeptide chain is converted into its native folded protein with the help of molecular chaperones. (**B**) Nascent polypeptide chain with a mutation (blue ball) folds into its native-like protein (or partial unfolded protein). (**C**) Mutant (or partial unfolded) protein may be re-recognized as imperfect proteins, leading to ubiquitination by E1 (ubiquitin activating enzyme), E2 (ubiquitin conjugating enzyme), and E3 (ubiquitin ligase). (**D**) Misfolded protein enters into the proteasome system with the help of the ubiquitin complex. (**E**) Misfolded protein is degraded into small peptide by proteasome and ubiquitin is regenerated. (**F**) Impaired proteasome system could not degrade the misfolded protein. (**G**) Native protein molecule is converted into misfolded structure, which is caused by destabilization of the α-helical structure and the simultaneous formation of the β-sheet structure. (**H**) Mutations accelerate protein misfolding. (**I**) Molecular chaperones facilitate to direct the misfolded proteins to the proteasomal pathway. (**J**) Misfolded monomers aggregate into dimer as initial building blocks for the formation of amyloid fibrils. (**K**) These building blocks further polymerize to form oligomers. (**L**) Molecular chaperones disaggregate the compact aggregates and develop native folded monomer. (**M**) Oligomers further form photofibrils. (**N**) Amyloid hypothesis. (**O**) Channel hypothesis.

The misfolded protein may lead to harmful effects, which can be divided into two categories (see Fig. 1 for details): (i) gain of function as observed in AD, Huntington’s disease, Parkinson’s disease, and Prion’s disease and (ii) loss of function as in the cases of CF and α1-antitrypsin deficiency.^1^ In the former category, the misfolded proteins may further aggregate to form amyloids, which finally cause cell toxicity and eventually death. More than twenty proteins in humans are involved in aberrant aggregation, include Aβ, prion, β2-microglobulin, tau, α-synuclein, etc. Aggregation can also occur in proteins with native conformations, although mutations usually accelerate this process. In other words, mutations are not an absolute requirement for protein misfolding and diseases. In the latter category, mutations are associated with defect in folding, leading to the accumulation of intermediate structure in endoplasmic reticulum (ER), as a consequence of increasing ER stress.^2^ Moreover, such mutations lead to the absence of the correctly folded proteins, resulting in the loss of some physiologically important functions.

## Protein Quality Control System: Molecular Chaperones and Ubiquitin Proteasome System

Proteins that are not able to reach their native states are recognized as misfolded and subsequently targeted to a degradation pathway (Fig. 1C). This is referred to the “protein quality control” system, which plays a critical role in cell function and survival and consists of two components: molecular chaperones and ubiquitin-proteasome pathway (UPP).^3^

Many molecular chaperones aid in normal folding of proteins and also in refolding of the abnormal conformations back to their native states, thus prevent the formation of misfolded or aggregated structures (Fig. 1A and L) and slow, arrest, or revert disease progression.^1^ They are capable of distinguishing between the native and non-native states of the targeted proteins. However, until now, it is still unclear how they distinguish between the correctly and incorrectly folded proteins and how they selectively target the latter for degradation. Molecular chaperones further facilitate to direct the misfolded proteins to the proteasomal pathway (Fig. 1I). Abnormal proteins can also be targeted to proteasome for degradation by covalent attachment to polyubiquitin, which is an energy-requiring process (Fig. 1C).^4^

However, in some cases, the protein quality control system is imperfect (Fig. 1F). There are two types of protein quality control defect, which have been linked to the etiology of an increasing list of congenital and acquired conformational diseases. In the first type, it is possible that the misfolded proteins are stable folding intermediates, thus they can escape from the degradation pathway and accumulate as aggregates, resulting in the formation of fibrilar deposits known as amyloids. In the second type, proteins harboring modest mutations not compromising their functional integrity may be recognized as misfolded, resulting in unnecessary degradation and consequently leading to the loss of functional phenotypes.

## From Protein Misfolding and Aggregation to Amyloid Fibrils

### Protein misfolding and aggregation

Protein misfolding and its pathogenic consequences have become an important research topic over the past decades. It is now well known that the molecular basis of protein aggregation into amyloid structures involves the existence of the misfolded forms of proteins (Fig. 1G and H). However, the critical problem is what factors are responsible for protein conformational changes leading to the misfolded forms. Over the past few years, a number of factors, such as mutations that destabilize the folded structure (Fig. 1H), changes in the environmental conditions (pH, oxidative stress, and metal ions), and the activity of certain proteins collectively named pathological chaperones (apolipoprotein E, amyloid P component, and protein X), have been identified to play such a critical role.^5^ Once a certain concentration of the misfolded protein is reached, the formation of their aggregates can occur in the cells (Fig. 1J and K), leading to the formation of an amyloid-like structure, which eventually causes different types of neurodegenerative disorders and ultimately leads to cell death.^3^

The formation of an aggregate is a common feature of all protein conformational diseases (PCDs). It is caused by the destabilization of the α-helical structures and the simultaneous formation of the β-sheet structures (Fig. 1G and H).^6^ These β-sheet structures are formed between alternating peptide strands. Linkages between these strands result from hydrogen bonding between their aligned pleated structures.^7^ Such β-linkages with a pleated strand from one molecule inserting into a pleated sheet of the next lead to the formation of intermolecular hydrogen bonding networks.

Before oligomerization, the fibrillogenesis often starts with dimers, which have been recognized as the initial building blocks of amyloid fibrils (Fig. 1J).^8^ They further polymerize into oligomers (Fig. 1K). During this nucleation process, the content of the secondary β-structure of the oligomeric assemblies is generally increased.^9^ After the nucleation or seeding step, the growing assemblies, ordered prefibrillar aggregates or protofibrils (Fig. 1M), are formed via an elongation process and eventually give rise to mature amyloid fibrils.^10^

### The relationship between protein misfolding and aggregation

Until now, it is still not clear whether conformational changes induce protein oligomerization and whether misfolding triggers protein aggregation.^11^ Based on the kinetic model of protein aggregation, it has been proposed that the critical event in PCDs is the formation of protein oligomers that can act as the seeds to induce protein misfolding. In this model, misfolding occurs as a consequence of aggregation (polymerization hypothesis),^11^ which follows a crystallization-like process dependent on nucleus formation.

An alternative model indicates that the underlying protein is stable in both the folded and misfolded forms in solution (conformation hypothesis).^5^ It suggests that spontaneous or induced conformational changes result in the formation of the misfolded protein, which may or may not form an aggregate. Several factors, such as those mentioned above, may cause the protein conformational changes and further lead to the misfolded forms of proteins.

In the third hypothesis, the native proteins undergo slightly conformational changes, resulting in the formation of an unstable amyloidogenic intermediate in the cellular environment. This unstable intermediate exposes many of its hydrophobic regions, leading to the growth of small oligomers, which mainly consist of β-sheets via intermolecular interactions. These small oligomers further form an ordered fibril-like structure named amyloid also via intermolecular interactions. In this model, the conversion of the folded protein into the pathological form is triggered by conformational changes, but the complete misfolding is dependent on oligomerization.^11^ So far, most of the experimental findings can be explained by the above three models. However, the conformation/oligomerization hypothesis is still the most comprehensive and accepted model for protein misfolding and aggregation.

### Amyloid aggregates lead to cell toxicity

Two hypotheses have been proposed to explain how the aggregates cause toxic effects to the cells (Fig. 1N and O). The first one, known as the “amyloid hypothesis” (Fig. 1N),^9^ directly believes that the huge amount of aggregates may damage organs simply by hindering a proper flow of nutrient to the cells, thus impairing tissue functions in the case of the peripheral amyloidosis. Recent studies have reported that an important role of modification to the intracellular free calcium and reactive oxygen species (ROS) occurs in cells exposed to toxic aggregate.^12^ It suggests that the oxidative stress following exposure to the early species involved in amyolid formation could damage cells and eventually cause cell death. However, it is still not clear why protein aggregation is followed by the production of ROS.

The second one, the so-called “channel hypothesis” (Fig. 1O), has been proposed to explain the biochemical mechanism of amyloid toxicity.^13^ This hypothesis also supports the changes of intracellular ion content and ROS state mentioned above. Protofibrils, the precursors of longer protofilaments and mature fibrils, typically appear as globular assemblies (2.5–5 nm in diameter) with a high β-sheet content and spontaneously organize into beaded chains and variously sized annular rings comprising small “doughnut” shaped species forming a central pore in membranes.^14^ It is still not possible to detect these channels in the cells involved in amyloid diseases due to the technical difficulties, but channels have been observed *in vitro* from a number of amyolid peptides and proteins, including islet amyloid polypeptide (IAPP or Amylin), a neurotoxic fragment of the prion protein (PrP_106–126_), serum amyloid A, polyglutamine, transthyretin (TTR), α-synuclein, and lysozyme. The observation of channels amongst such a diverse variety of peptide sequences suggests a deep underlying similarity in their physical chemical structures. This special amyloid pore may account for the toxicity of the amyloid aggregates. Thus, protein oligomers may act as a biological signal killing the target cells by forming non-specific membrane pores, which further result in the unbalance of the ion concentration.

## Toxic Amyloid Formation Causes Many Human Neurodegenerative Disorders: The Cases of AD and Prion’s Disease

AD and prion disease are the primary and frequent discovered diseases involving selective neuronal vulnerability with degeneration in specific brain regions and deposits of the corresponding misfolded protein in neurons and other cells. In this section, we aim to elucidate the causes of human neurodegenerative disorders through the formation of toxic amyloids by the cases of AD and Prion’s disease.

### Alzheimer’s disease

AD is a progressive neurodegenerative disease characterized by extracellular amyloid plaques and intraneuronal fibrillary tangles in the brain. It has been shown that Aβ_1–40_ and Aβ_1–42_ are the main alloforms of amyloid β (Aβ) peptides found in amyloid plaques. Aβ peptides are derived from proteolytic processing of the amyloid precursor protein (APP). APP can be cleaved by three different proteases, named α-, β-, and γ-secretases. In general, β-secretase cleaves the extracellular domain of APP to generate the N terminus of Aβ, while γ-secretase performs proteolysis in the middle of the transmembrane domain of APP to produce the C terminus of Aβ.^15^ Finally, the two main products, Aβ_1–40_ and Aβ_1–42_, migrate outside the cell and give rise to fibrils.

Several mechanisms have been proposed to explain the neuron toxicity of Aβ peptides, such as the formation of ion channels on the cell membrane,^16^ the generation of free radicals,^17^ and the interaction of Aβ peptides with various receptors, such as apolipoprotein E^18^ and mitochondrial hydroxyacyl-CoA dehydrogenase.^19^ However, the cytotoxic mechanism of Aβ has not yet been fully understood at present. Compelling evidences have demonstrated that the soluble oligomers (amyloid-derived diffusible ligands; ADDLs) and fibrils of Aβ are the toxic identities that cause neuronal injury and death in patients suffering from AD.^20^

### Prion’s disease

The prion protein is thought to cause a disease in cattle called bovine spongiform encephalopathy (BSE) or “mad cow disease” and a disease in human named variant Creutzfeldt-Jakob disease (vCJD).^5^ It is known that normal prion protein is protease sensitive, soluble, innocuous, and has a high α-helical content. This protein is thought to undergo a conformational change in which α-helices of the wild-type protein PrP^C^ (normal cellular form) are converted into β-sheet-dominant PrP^SC^ (pathogenic isoform), resulting in misfolding and aggregation. PrP^C^ and PrP^SC^ share the same covalent structure but possess different folds. PrP^SC^ has been identified as the causative agent in transmissible spongiform encephalopathies because it is capable of aggregating into a variety of forms from amorphous to highly structured aggregates.

A central theme in prion disease research is the detection of the process underlying the conformational transition from PrP^C^ to PrP^SC^. PrP^C^ has been found to undergo a pH-dependent conformational change in the range of pH 4.4–6.0, with a loss of α-helical content and a gain of β-structure.^21^ PrP^SC^ also acts as a template for the structural conversion of PrP^C^ as well as huntingtin proteins, subsequently forming aggregates.^22^ Moreover, unlike AD, it is believed that in Prion’s disease, the conformational infection and aggregation can take place both extracellularly and intracellularly.^23^

## Protein Misfolding with the Loss of Function Lead to Several Lethal Diseases: The Case of CF

The human cystic fibrosis transmembrane conductance regulator (CFTR) gene encodes an integral membrane glycoprotein of 1,480 amino acid residues with two N-linked glycosylation sites.^24^ The CFTR is a cAMP-regulated chloride (Cl^−^) channel localized at the apical membrane of secretory epithelia. Mutations in this channel cause CF, a disease characterized by the inability of epithelial cells to secrete chloride, result in the production of thick and viscous mucus that causes severe functional obstruction of lungs and pancreas. A majority of CF patients has a deletion of a phenylalanine residue at position 508, resulting in an F508del-CFTR protein.^25^ The clinical importance of this mutation becomes evident because it accounts for 90% of patients diagnosed with CF.^26^ This mutation results in a misfolded channel retaining in the ER in an immature state and are rapidly degraded by a process involving the ubiquitin-dependent proteasomal system.^24^ Thus very little of this protein can reach the cellular membrane, resulting in the loss of function phenotype.

## Potential Treatments for PCDs

Drugs against PCDs in humans aim to inhibit aggregation and/or to enable the mutant proteins to escape from the protein quality control systems so that their function can be rescued. Recently, several low-molecular-weight compounds, named chemical chaperones,^27^ have been shown to act as the potential therapeutic agents for the control of many PCDs. In this section, we provide some evidences showing that chemical chaperones or inhibitors can be used as potential therapeutic agents for the control of several PCDs in humans, such as AD, Prion’s disease, and CF.

### Potential treatment for AD

Aβ has emerged as the most promising target in the treatment or prevention of AD. Inhibition of Aβ-fibril formation might be a reasonable therapeutic strategy because familial mutations that lead to an increase in Aβ concentration or to its aggregation increase neuropathology.^28^ Unfortunately, no effective therapy using a chemical chaperone system has been successfully conducted so far. A previous study has shown that osmolytes such as glycerol and trimethylamine *N*-oxide (TMAO), acting as chemical chaperones, correct folding defects by preferentially hydrating partially denatured proteins and entropically stabilize native conformations.^29^ Such information could potentially be used to develop cellular models of Aβ aggregation and the assessment of agents that modulate fibril formation. In this case, chemical chaperones could exacerbate the pathophysiologic state. However, a variety of chemical compounds have been found to inhibit the fibrillation of Aβ, including antibiotics, benzofuran derivatives, sulfonated dyes, styryl benzene, flavone, and peptidic β-sheet breakers. Cell culture experiments have indicated that Aβ inhibitors could reduce the cytotoxicity of Aβ peptides and the amount of amyloid deposition in mice with acute amyloidosis.^30^ However, very few of them have entered into clinic trial phases because of the problems inherent in these inhibitors, including low bioavailability, poor biostability, toxicity, and inability across the blood-brain barrier (BBB), limiting their therapeutic applications. Thus, structural modification of inhibitors as well as design of new inhibitors with alternative structural scaffolds is necessary to improve the physiochemical properties of these compounds in the future.

### Potential treatment for Prion’s disease

Therapeutic agents are often designed in an attempt to destroy the PrP^SC^ structure and hopefully recovery the PrP^C^ structure or any other innocuous isoform. For example, several β-sheet breaker peptides, which could slow or reverse disease progression, have been designed to disrupt the β-rich amyloidgenic PrP^SC^ species. However, it has been shown that disrupting PrP^SC^ is not sufficient to inhibit Prion’s disease and that this strategy may increase propagation of PrP^SC^ and infectivity.^31^ Thus, strategies for stabilizating the PrP^C^ conformation also appear as alternative approaches.

Some chemical chaperons, such as TMAO, dimethyl sulfoxide (DMSO), and glycerol, are thought to stabilize the PrP^C^ conformation and have been suggested to be effective in the destruction/protection strategy. In a previous study, Tatzelt and co-workers have shown that TMAO and other protective osmolytes successfully prevent scrapie formation *in vitro*.^32^ Moreover, a number of other compounds, such as anthracyclines, porphyrins, and diazo dyes, have also been shown to inhibit prion replication when administered with PrP^SC^ in animal models.^33^ It is regretful that this is not a clinically relevant model for therapeutic intervention because subclinical disease exists for months in mice and years in humans.^7^ However, a recent study by Vogtherr and co-workers has suggested that quinacrine binds specifically to PrP^C^ and inhibit the conversion of PrP^C^ to PrP^Sc^, which in turn suppresses the progression of Creutzfeldt-Jacob disease.^34^ Currently, quinacrine has been clinically approved and clinical trials are being carried out to test the usefulness of this molecule in patients suffering from Creutzfeldt-Jacob disease.

### Potential treatment for CF

There are numerous ongoing efforts towards finding agents that promote the folding or block the degradation of nascent F508del-CFTR with the potential to provide a therapeutic basis for the treatment of CF. For example, Sato et al. have examined the effect of glycerol on the fate of the mutant F508del-CFTR and concluded that 10% glycerol (vol/vol) could mediate an increase in the transport of the mutant protein from the ER to the plasma membrane in a cell culture model, which is associated with an increase in the functional activity of CFTR.^35^ In addition, two other low-molecular-weight organic compounds, deuterated water and TMAO, have also been shown to increase the post-translational maturation of F508del-CFTR in a cell culture model, leading to the increase of the chloride transport activity.^36^ Moreover, other study has also reported that chloride transport is increased significantly by the use of DMSO on polarized epithelial cell lines expressing F508del-CFTR.^37^ These results all suggest that these low-molecular-weight compounds could stabilize this mutant, promoting the proper folding and transport to its site of function.

### The role of chemical chaperones in the treatment for PCDs

Until now, the precise mechanisms of the action of chemical chaperones are still not fully understood. However, they are thought to provide positive effect on protein folding, to reduce protein aggregation, and to stabilize a conformation capable of escaping from the degradation pathway. For example, osmolytes, such as glycerol, are believed to stabilize the native structures of proteins, which can be explained by their preferential exclusion from the surfaces of proteins. This effect increases the chemical potential of the protein and is proportional to the solvent-exposed surface area of the protein, thus more expanded conformations are disfavored in the presence of osmolytes and the protein tends to adopt the more compact native structure.^38^ Recently, Carprnter et al. have investigated the effects of sucrose, a model osmolyte, on the conformational equilibria and fluctuations within the native-state ensembles of bovine pancreatic ribonuclease A and S and horse heart cytochrome *c* and concluded that the presence of sucrose shifts the conformational equilibria toward the most compact protein species within the native-state ensembles due to the preferential exclusion of sucrose from the protein surface.^39^

Besides the human diseases and their corresponding therapies mentioned above, lysosomal storage diseases have been tested for possible chemical chaperone therapy in humans. For example, Matsuda et al. have synthesized a compound, *N*-octyl-4-epi-β-valienamine (NOEV), for molecular therapy of brain pathology in β-galactosidosis and confirmed its restorative effect on the model mouse brain after short-term oral administration.^40^ Moreover, Tropak and coworkers have shown that increasing the amount of hexosaminidase A is capable of exiting the endoplasmic reticulum for transport to the lysosome and propose that such hexosaminidase inhibitors can function as pharmacological chaperones by enhancing the stability of the native conformation of the enzyme.^41^

However, these chemical chaperones are a far from practical therapeutic approach in humans because the necessary efficacious dose is toxic. Nevertheless, exploiting the mechanism by which they are effective may yield clues for the design of new compounds, which are less toxic. For example, Zhang et al. have reported that some organic solutes, such as *myo*-inositol, betaine, and taurine, exhibit the ability to restore the folding defect of F508del-CFTR.^42^ Despite their reduced toxicity in comparison to glycerol, the robustness and corrective efficacy of such compounds remain to be demonstrated in the whole organisms. Encouragingly, some *in vivo* data has already shown that such strategy may be valid.^43^ Moreover, recent study has reported that various disaccharides can inhibit the polyglutamine-mediated protein aggregation.^44^ Tanaka et al. have found that trehalose, the most effective disaccharides, can decrease polyglutamine aggregates in cerebrum and liver due to the improved motor dysfunction and extended lifespan in a transgenic mouse model of Huntington disease.^44^ Most importantly, trehalose is nontoxic with high solubility and can be coupled with efficacy upon oral administration, which makes trehalose a promising therapeutic drug or a leading compound for the treatment of polyglutamine diseases.^44^ These evidences together suggest that chemical chaperones are potential pharmaceuticals for the treatment of protein misfolding diseases.

## Insights into the Process of Protein Misfolding: Computational Studies

Understanding the misfolded structure of the amyloid-associated proteins and how they change their conformations to the misfolded and/or toxic forms can help to elucidate their aggregation mechanisms and may contribute to the development of some effective therapies for treating PCDs in humans. Drug compounds are generally designed to inhibit, restore, or otherwise modify the structure and behavior of the disease-associated proteins. Target proteins are typically the key molecules involved in a specific metabolic or cell signaling pathway that is known or is believed to be related to a particular disease state.

The early stage in drug discovery involves target discovery, validation, and identification by high-throughput physical and/or virtual screening. So far, structure-based drug design is considered as one of the most powerful approaches in drug discovery platform and ca be applied computationally when the structure of the target protein is (i) known based on the crystallographic, nuclear magnetic resonance (NMR) techniques, or other experimental methods, or is (ii) unknown yet to be built using homology modeling. The process often involves the generation of a very large *in silico* library of potential derivatives and the use of molecular docking to select derivatives that may interact with the target protein on the basis of shape complementarities and charge placement.

However, the critical problem is the structural information about the target protein must be available. Unfortunately, due to the difficulties in crystallizing amyloid fibrils, the detailed intrinsic structure has yet to be determined by x-ray diffraction. Thus, some biophysical techniques, such as transmission and cryo-electron microscopy, atomic force microscopy (AFM), and solid-state NMR, have been used to disclose the structural features of amyloid fibrils. Nevertheless, these techniques are still difficult to provide structural information in atomic detail. For the case of Aβ, some evidences have suggested that Aβ exists as a mixture of α-helix, β-sheet, and random coil in aqueous solution.^45^ However, in fluorinated alcohols, such as trifluoroethanol (TFE) or hexfluoro 2-propanol (HFIP), it adopts a stable α-helical conformation.^46^ It is known that the α-helical conformation of Aβ is temperature sensitive but its β-sheet conformation is not. Moreover, the α-helical conformation of Aβ is favored at pHs 1–4 and 7–10; whereas the β-sheet conformation of Aβ is favored at pH 4–7.^47^ X-ray diffraction analyses and NMR determinations have reported that Aβ stacks in the form of pleated β-sheet structures oriented perpendicular to the fibril axis.^48^ Furthermore, several conformational studies on Aβ fibrillogenesis have suggested that the conformational transition of Aβ into β-sheet structure in fibrils goes through an α-helix-containing intermediate conformation.^49^ Thus, it is extraordinarily difficult to design an inhibitor due to the structural complexity of Aβ. Therefore, computational studies provide an alternative tool to elucidate the transition between the unaggregated and aggregated proteins, where experimental techniques cannot yet probe.

Molecular dynamics (MD) simulation has been employed as a powerful tool to provide structural information in atomic detail under various conditions. For the case of Aβ, numbers of MD simulations have been performed by several groups with a view to elucidating the conformational transition and assembly mechanism based on the modeled structures of Aβ, i.e. fragments or synthesized analogs of Aβ. For example, Klimov and Thirumalai have performed a series of MD simulations towards Aβ peptide and their results showed that Aβ_16–22_ peptides form antiparallel β-sheet structures and the α-helical intermediates are transiently populated.^50^ They further proposed that fibril formation by Aβ peptides, which occurs by maximizing the number of slat bridges and hydrophobic interactions, must involve oligomers with high α-helical content. In a more recent study, atomic detail MD simulations with explicit solvent have been conducted to show that only large Aβ_16–22_ oligomers with at least 8–16 monomers form a stable β-sheet aggregate through better hydrophobic contacts and a better shielding of backbone-backbone hydrogen bonds from the solvent.^51^ In the case of prion, two 10-ns trajectories generated by MD simulations have been used to probe the initial events in the conformational transition of PrP^C^ to the aberrant aggregation-prone form.^52^ More recently, DeMarco and Daggeat have simulated the fragment of human prion protein (residues 90–230) at low pH, which triggers misfolding of this protein, and observed a conformational transition to a PrP^SC^-like isoform.^53^ Their results further demonstrated that the N-terminal portion of this protein, which has been identified as a probable aggregation site, undergoes extensive conformational rearrangement, leading to the formation of a large solvent-accessible hydrophobic cluster.

**Figure 2.**
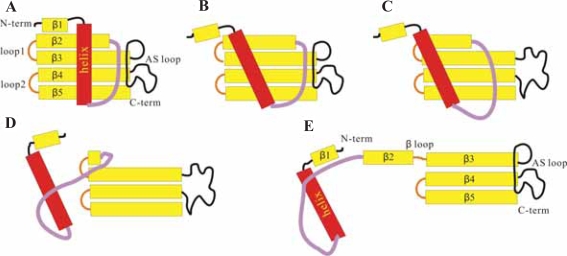
The proposed mechanism of the 3D domain swapping process of HCC. (**A**) The closed form of the monomeric HCC;^52^ (**B**) partially unfolded monomeric HCC with the central α-helix moving away from the β-region via the disruption of the interior hydrophobic core; (**C**) partially unfolded monomeric HCC with the disappearance of the native contacts between β2 and β3-AS; (**D**) partially unfolded monomeric HCC with the β2-L1-β3 hairpin unfolded following the “zip-up” mechanism; and (**E**) the open form of the monomeric HCC.^54^

In addition to prion protein and Aβ peptide, other amyloid-associated proteins have also been investigated intensively by MD simulations. For example, TTR has been shown to be the cause of or involved in senile systemic amyloidosis.^54^ From the MD simulation results, Armen et al. have proposed that the formation of the “α-sheet” structure by alternating α_L_ and α_R_ residues may represent a key pathological conformation during amyloidogenesis.^55^ Another case is human cystatin C (HCC), which is thought to co-localize with Aβ peptide in the dimeric or oligomeric form in brain amyloid deposits of patients, particularly in elderly individuals and patients suffering from AD or Down’s syndrome.^56^ The structure of HCC consists of a core with a five-stranded anti-parallel β-sheet (β-region) wrapped around a central helix.^57^ The monomeric HCC is thought to form a dimeric structure through the so-called “3D domain swapping” process.^58^ The results from our previous MD study has allowed us to propose a possible mechanism for HCC domain swapping as follows: (i) first, the central helix departs from the β-region via the disruption of the interior hydrophobic core (Fig. 2A,B); (ii) subsequently, the native contacts within β2 and AS loop disappear (Fig. 2B,C); (iii) then, the β-hairpin between β2 and β3 unfolds through the destruction of three important salt bridges following the so-called “zip-up” mechanism (Fig. 2C,D); and (iv) finally, the open form of the monomeric HCC is generated (Fig. 2E).^59^

Most importantly, the results of the above mentioned MD simulations are all in good agreement with the available experimental observations, providing atomic insights into the conformational changes associated various PCDs in humans and may contribute to the development of structure-based drug design.

## Possible Therapeutic Strategies for PCDs: Combining MD Simulations and Structure-Based Drug Design

Recently, a combined approach of MD simulation and structure-based drug design was conducted by Liu and co-workers.^60^ Long time MD simulations were conducted to investigate the conformational transition of Aβ_1–40_ and their results showed that Aβ adopts a α-helix/β-sheet intermediate structure, which exhibits a core domain constituted by the segment of residues 24–37. In their later study, they performed virtual screening based on molecular docking towards this Aβ intermediate structure and aimed to design inhibitors, which can bind to the β-sheet region of the Aβ intermediate to interrupt the formation of the pleated β-sheet structure found in amyloid fibrils.^55^ From the results of thioflavin T fluorescence assay and AFM determination, they have successfully identified a new inhibitor, named as DC-AB1, to abolish Aβ fibrillation. This study not only reveals some clues to understanding the molecular events involved in Aβ aggregation, but also provides a strategy for inhibitor design based on the flexible intermediate structures of Aβ peptides.

From their promising results, we can expect that the combined experimental and computational approaches to design potential interfering compounds for inhibiting protein aggregation associated with PCDs in humans will increase dramatically in the near future of drug discovery and development history.

## Conclusions

In this review, we discussed the mechanisms of various PCDs in humans, including AD, Prion’s disease, and CF, and the strategies of using chemical chaperones and inhibitors as therapeutic agents against these diseases. To further understand the biological meanings and aggregation mechanisms of either amyloid-associated proteins or normal proteins with the ability to aggregate into amyloid fibrils, their structures in atomic detail must be available. However, the lack of the detailed structural information of amyloid fibril makes it difficult to elucidate their aggregation mechanisms. At this stage, MD simulation combined with some experimental approach may provide a powerful tool to gain the atomic insights into the possible mechanisms of protein misfolding and aggregation associated with a number of PCDs in humans. Thus, it may further assist the structure-based drug design in the near future of drug discovery and development history.
